# Editorial: The Implications of Weight Bias Internalization

**DOI:** 10.3389/fpsyg.2019.03019

**Published:** 2020-01-14

**Authors:** Stuart W. Flint, Jayne Raisborough, Joanne Hudson

**Affiliations:** ^1^School of Psychology, University of Leeds, Leeds, United Kingdom; ^2^Scaled Insights, Nexus, University of Leeds, Leeds, United Kingdom; ^3^School of Cultural Studies and Humanities, Leeds Beckett University, Leeds, United Kingdom; ^4^Applied Sports Technology and Exercise Medicine Research Centre, Swansea University, Swansea, United Kingdom

**Keywords:** weight bias and stigma, obesity, weight, weight bias internalization, weight discrimination, policy, public health policy

## Introduction and Edition Purpose

Weight stigma and discrimination have become a topic of global importance. Indeed, this is underscored by the evidenced impact of these experiences on physical and mental health and health related behaviors such as avoidance of healthcare environments and reduced healthcare seeking behaviors (e.g., Puhl and Suh, 2015). Extant research (e.g., Latner et al., 2013) has focused on weight bias internalization (WBI) and the associated implication, which include reduced quality of life and maladaptive behavioral response. WBI refers to “internalization of negative weight stereotypes and subsequent self-disparagement” (Pearl and Puhl, 2018, p. 1141). Although people across the weight spectrum experience WBI, it is most commonly experienced by people with a higher weight status. The commonality of weight stigma and discrimination experiences for people with overweight and obesity means that internalization of weight bias is likely, and, with the associated impacts on health and health behaviors, is an important consideration across society.

In editing this Research Topic, we have sought to present emerging empirical and theoretical contributions that advance current understanding of the impact of WBI, specifically on health and health behavior and the underlying mechanisms that lead to WBI. Thus, we present a range of research contributions from across the world, which hold important implications for policymakers and healthcare practitioners.

## Summary of contributing Articles

First, Noonan-Gunning presents a critical qualitative exploration to understand parents' lived experiences of food policy and resistance to stigma. Her empirical study demonstrates how stigma and resistance develop as a response to policy. With specific attention to parents' interactions with child policy, Noonan-Gunning provides insight into the interaction of notions of responsibility and morality.

The second article is a two-study investigation of the impact that body size has on daily life activities of women with obesity. First, using ethnographic techniques and interviews based on video recordings, Urdapilleta et al. provide in-depth information about the behaviors of women with, or previously with, obesity in response to embarrassing experiences related to body size and stigma. Second, Urdapilleta et al. reported that in a mirrored condition, women with obesity overestimated their body size by 30%, and that estimations of body size were least accurate for women who had bariatric surgery.

The third article presents a narrative inquiry of weight bias and obesity stigma to explore the experiences of people living with obesity, in order to develop counter stories and identify opportunities for change. Ramos Salas et al. presented 10 counter stories to personal, familial, professional, and social contexts where people living with obesity experience weight bias and obesity stigma. They reported that internalization of weight bias led to emotional responses including shame, depression and suicidal thoughts and actions. They also argue that WBI led to maladaptive responses including healthcare avoidance. When working with individuals with obesity, the authors highlighted the importance of developing self-compassion and self-acceptance, whilst resisting damaged social identities and demanding respect, dignity, and fair treatment.

Meadows and Higgs examined the impact of WBI on objectively measured food intake. After completing a batch of questionnaires, participants read either a bogus news article on the negative consequences of weight or smoking before 15 min exposure to a selection of sweet and savory snacks. The authors reported that internalization of weight bias did not predict total energy intake. They did however, find that participants of higher weight, who had high levels of WBI, consumed fewer snack calories after reading the news article about the negative effects of weight compared to reading an article focused on negative effects of smoking. No effects were observed for participants of normative weight.

Williams and Annandale provide an opinion article that aims to broaden the way that internalization is defined and analyzed in weight stigma research, purporting that this will increase understanding of the implications of WBI. As such, Williams and Annandale challenge the current application of internalization terminology, arguing that it is largely embodied, and therefore to fully understand the implications of WBI, an understanding of how and in what ways these experiences “get under the skin” is warranted.

Essential to research exploring the WBI is the quality of measures. The Weight Bias Internalization Scale is an 11-item measure, developed from an original pool of 19-items. The original scale was created based on a unidimensional structure, however Meadows and Higgs postulated that there is a multi-dimensional nature to WBI. To explore this, they conducted an exploratory and confirmatory analysis of the original 19-items. They reported that the internalization of weight bias is a multi-dimensional concept where two-factors of the WBI scale are suggested for use to explore the relationships between different aspects of internalized weight bias.

Whilst evidence highlights that experiences of WBI are associated with reduced global quality of life, less is known about weight specific domains of quality of life. Walsh et al. recruited 178 adults with obesity from a weight loss trial, who completed measures of WBI, weight specific domains of quality of life, and, patient health and depression. Walsh et al. reported a relationship between WBI and mental and physical aspects of weight-related quality of life, independent of any effect of gender or race. This study provides further evidence to highlight the need to end weight stigma and discrimination, and given the commonality of such experiences, consider the effects of internalization of weight bias within healthcare.

In another study seeking to extend the current evidence base around the impact of WBI and mental and physical health, Puhl and Himmelstein conducted a cross sectional study recruiting adolescents seeking weight loss treatment. The authors reported that both male and female participants seeking weight loss treatment had high levels of WBI, and that higher levels of WBI were reported by adolescents engaging in binge eating and eating to cope with distress. They also found that mothers' weight-related comments and dieting frequency predicted adolescents' WBI. This study highlights the potential impact of parent communication about weight and dieting behavior for adolescents and their families seeking weight loss treatment.

Finally, Täuber et al. compared the impact of framing overweight and obesity as incompetence or immoral. First, Täuber et al. experimentally compared exposure to weight stigma framed as immoral vs. incompetence. They reported that people with overweight and obesity respond by defending their moral social-image but that this is less effective for encouraging weight loss. Exposure to weight stigma framed as incompetence led to an increased likelihood of engagement in weight loss behaviors. Second, they examined the notion that WBI primarily revolves around moral concerns, which is likely to lead to less self-determined behavioral regulation. They found that WBI was associated with less self-determined and more other-determined regulation of dieting and exercising. This suggests that WBI leads to maladaptive behavioral regulation, contributing to lower psychological functioning and well-being of people with overweight and obesity.

## Emergent Recommendations

We offer several recommendations. First, that when healthcare professionals work with people living with obesity considerations are made for WBI and that efforts are made to reduce this internalization given that it is a key contributor to mental and physical health concerns and is associated with maladaptive health behaviors. This could include standardized/compulsory training and improved educational resources. Second, that policymakers, media and other disseminators of information avoid the moralization of weight given its debilitating effect on health and health behavior. Third, that researchers explore methods to reduce WBI and identify coping methods that could be employed both in society and healthcare environments.

## Author Contributions

SF, JR, and JH drafted, revised, and finalized the content of the manuscript. All authors have read and approved the final manuscript.

### Conflict of Interest

The authors declare that the research was conducted in the absence of any commercial or financial relationships that could be construed as a potential conflict of interest.
